# An update of COVID‐19 pandemic in India

**DOI:** 10.1002/hsr2.359

**Published:** 2021-08-31

**Authors:** Kumudhaveni B, Thirumal M, Radha R

**Affiliations:** ^1^ Department of Pharmacognosy, College of Pharmacy Madras Medical College Chennai Tamilnadu India; ^2^ Department of Pharmacognosy, SRM College of Pharmacy SRM Institute of Science and Technology Chennai Tamilnadu India

## 1 INTRODUCTION

A severe pneumonia case was reported in Wuhan, Hubei province on December 8, 2019. China informed World Health Organization (WHO) on December 31, 2019, about the detected pneumonia cases of unknown aetiology in Wuhan.^1^ A novel strain of coronavirus was isolated on January 7, 2020, which is similar to the same family of viruses as severe acute respiratory syndrome (SARS) and the Middle East respiratory syndrome (MERS).^2^ This virus was a positive, enveloped, single‐strand RNA virus, and the disease was named COVID‐19, “CO” stands for corona, “VI” stands for virus and “D” stands for disease. China declared officially outbreak as an epidemic on January 20, 2020 after findings of human‐to‐human transmission.^3^ On January 31, WHO declared a public health emergency after a worldwide death of more than 200 and more than 9800 cases. On March 11, 2020, WHO declared COVID‐19 a global Pandemic after the spread and severity of the outbreak.^4^

## 2 SUBJECTS

### 2.1 Symptoms

The virus is transmitted from the infected person through respiratory droplets and by touching the surfaces, contaminated with viruses. Symptoms of coronavirus are fever, headache, cold, cough, body pain, diarrhea, vomiting, tiredness, loss of taste and smell, shortness of breath, breathing difficulties, or pneumonia. To avoid the infection, WHO advised to follow the steps include washing the hands using soap and water for at least 20 seconds or with alcohol‐based hand rub, wearing face masks to cover nose and mouth, maintaining social distance, and seeking medical care if any symptoms occur.^5^

### 2.2 Cases in India

In India, the first case of COVID‐19 infection, a female of 20 years old was reported in Thrissur, Kerala, on January 30, 2020.^1^ Later number of cases was increased in India. Over 27 million cases and more than 300 000 deaths were reported in India. Then the peak was declined in September 2020. Since March 2021, India witnessed a massive second wave of COVID‐19 cases.^6^ As on July 26, 2021 08:00 am IST (GMT + 5:30), total cases across India are 31 411 262, discharged 30 579 106 and deaths 420 967.^7^ India's first COVID‐19‐infected female has again tested positive for COVID‐19.^8^

### 2.3 Variants

When the virus over time transmits in a huge population, it keeps on replicating leads to changes in the RNA of the virus called mutation and the virus is mutant of the original virus. Viruses with these mutations are called variants. Variants of SARS‐CoV‐2 are assuming threatening proportions to cause disease, death and escaping from immune response developed by vaccines. WHO classified variants into variants of interest, variants of concern (VOC) and variants of high consequences without attaching any countries name with the origin of the variants. Variants of interest are Epsilon:B.1.427, Zeta:P.2, Eta:B.1.525, Theta:P.3, Lota:B.1.526, Kappa:B.1.617.1, and Lambda:C.37. Variants lead to more severe form of the disease are called VOC. It includes Alpha:B.1.1.7, Beta:B.1.351, Gamma:P.1, Delta:B.1.671.2.^9^ Alpha variant was first identified in United Kingdom (2020), Beta was originated in South Africa (December 2020), Gamma was first identified in Brazil (October 2020), Delta was appeared in India (October 2020). Alpha has mutations N501Y, P681H, Y144/145, Beta has N501Y, K417N, E484K, Gamma has N501Y, K417T, E484K and Delta has E484Q, L452R mutations. Delta variant was responsible for sudden increase in COVID‐19 cases in India for the past few months. Currently, variant of high consequences is none.^9^, ^10^

In early February 2021, an outbreak of COVID‐19 occurred in Bengaluru was by related virus belonging to the B.1.36 lineage. The B.1.36 lineage was imported by international travel and circulating in Bengaluru city. The lineage was characterized by aminoacid replacements. The immune escape associated amino acid change, N440K has been found from states like Karnataka, Andhrapradesh, Maharashtra, and Telangana, and also associated with reinfection.^11^

### 2.4 Delta Plus variant

Delta Plus or Delta‐AY.1 variant, a sublineage of Delta variant, is a mutated version of B.1.617.2 variant. It is characterized by K417N mutation in the spike protein of COVID‐19 virus.^12^ Fifty‐six cases of Delta plus variant has registered in 12 states in India till June 30, 2021. Central Government declared Delta Plus variant as a Variant of concern in India.^13^

### 2.5 Diagnosis

Real‐time reverse transcription‐polymerase chain reaction test (RT‐PCR) is the most accurate method for testing COVID‐19. It takes 24 to 48 hours for generating the results. Nucleic acid amplification test (NAAT)‐based RT‐PCR is a gold standard test for confirmation of COVID‐19 cases. Serology‐based antibody testing, Immunoassays like ELISA, lateral flow immunoassay and chemiluminescent immunoassay help to detect Immunoglobulin G (IgG) and immunoglobulin M (IgM). Antigen‐based rapid tests, Chest X‐rays, Chest CT scan, high‐resolution computed tomography (HRCT) and commercially available RT‐PCR test kits are various diagnosis tests for COVID‐19. C‐reactive protein, D‐dimer and other blood tests, and stool samples are also used in the diagnosis of disease.^14^, ^15^

### 2.6 Latest treatment guidelines

On June 3, 2021, Food and Drug Administration (FDA) updated emergency use authorization of anti‐SARS‐CoV‐2 monoclonal antibody combination of Casirivimab 600 mg plus Imdevimab 600 mg for IV infusion. If IV infusion is not feasible, subcutaneous injection can be administered. Sotrovimab another monoclonal antibody products also be recommended for non‐hospitalized patients. Therapeutic management for high risk of disease hospitalized patient receive Remdesivir, Dexamethasone (use of Dexamethasone should not exceed 10 days). Based on the oxygen need, Toclizumab is also given. After hospital discharge, these drugs are not continually used. Ritonavir, Ivermectin, supplements such as vitamin C, vitamin D and zinc are recommended. FDA did not approve hydroxychloroquine, chloroquine for the treatment of COVID‐19. Low‐titer COVID‐19 convalescent plasma is not authorized. High‐titer COVID‐19 convalescent plasma is not recommended for nonhospitalized patients and hospitalized patients with impared immunity. Anti‐inflammatory, immunomodulatory therapy, injection methyl prednisolone, anticoagulant were advisable as per the patient's need.^16^

### 2.7 Traditional system of medicine treatment in India

Ministry of Ayush, Government of India has given guidelines for the treatment of COVID‐19. In the Siddha system of medicine, treatment for mild and moderate symptoms includes Kaba Sura Kudineer, Adathoda Manapagu, Nellikai Ilagam, Thalisathi Vadagam, Nilavembu Kudineer, and Thippili Rasayanam. In the Ayurveda system of medicine treatment includes Mahasudarshana Ghanvati, Sanjeevani Vati, Talishadi Churna, Lavangadivati, Trikatu Siddha Jala, Agasthya Rasayana, Chayavanaprashavleha, and Bramha Rasayana. In the Homoeopathy system of medicines, treatment includes *Aconite napellus, Arsenicum album, Bryoni alba, Ipecacuanha, Belladonna*, and *Camphora* were prescribed depending upon the symptoms.^17^

### 2.8 Vaccines for COVID‐19

Covishield and Covaxin vaccines are being administered in India. Covishield has been developed by Pune‐based Serum Institute of India and AstraZeneca‐Oxford University. Covaxin is manufactured by Bharat Biotech in collaboration with the Indian Council of Medical Research (ICMR) and the National Institute of Virology, India. The Drugs Control General of India approved Russia's Sputnik V COVID‐19 vaccine. Gurgaon, on July 10, 2021, became the first district to administer Sputnik V vaccine in the country.^18^ Total vaccination doses administered in India as on July 26, 2021 08:00 am IST (GMT + 5:30) is 435 196 001.^7^

## 3 CONCLUSION

Vaccines are critical tool in the battle against the COVID‐19 war. We need to use this tool to vaccinate even if the vaccines may be somewhat less effective against some of the COVID‐19 virus variants. Even if we are vaccinated, we have to take certain preventive measures like using face masks, often washing hands with soap water or rub with alcohol‐based sanitizers, and maintaining social distance until the COVID‐19 cases dropdown.

## CONFLICT OF INTEREST

All authors have no conflict of interest to declare.

## AUTHOR CONTRIBUTIONS

Conceptualization: Kumudhaveni B, Thirumal M

Writing – Original Draft Preparation: Kumudhaveni B

Writing – Review & Editing: Kumudhaveni B, Thirumal M, Radha R

Supervision: Radha R

All authors have read and approved the final version of the manuscript.

## TRANSPARENCY STATEMENT

Kumudhaveni B affirms that the manuscript is an honest, accurate and transparent account of the study being reported; that no important aspects of the study have been omitted; and that any discrepancies from the study as planned (and, if relevant, registered) have been explained.

## Data Availability

The authors confirm that the data that supports the findings of this study are openly available at https://www.mygov.in/covid-19.
